# Emergency Medical Service Responders’ Perspectives on Transgender, Intersexual, and Non-Binary Patients in Germany

**DOI:** 10.5811/westjem.39705

**Published:** 2025-05-19

**Authors:** Torben Brod, Kambiz Afshar, Christoph Schroeder, Carsten Stoetzer, Stephanie Stiel

**Affiliations:** * Hannover Medical School, Department of Emergency Medicine, Hannover, Germany; † Hannover Medical School, Institute for General Practice and Palliative Care, Hannover, Germany

## Abstract

**Introduction:**

Gender minorities, including transgender, intersexual and non-binary (TIN) individuals, are at risk of receiving suboptimal care in emergency departments due to clinicians’ limited knowledge and formal training in TIN-specific needs. Little evidence is available regarding emergency medical service (EMS) responders, including paramedics (EMT-P), emergency medical technicians (EMT) ,and prehospital emergency physicians (EP). Therefore, in this study we aimed to explore the experiences and knowledge, attitudes, and education/training needs of EMS professionals in Germany regarding the care of TIN patients.

**Methods:**

In April 2023, we electronically surveyed EMTs, EMT-Ps and prehospital EPs from ambulance stations across Germany. Participants completed a questionnaire consisting of 15 closed-ended items assessing their experience and knowledge, attitudes, and education/training needs regarding the care of TIN patients. We used standard descriptive statistics and tested for group differences using the chi-square test.

**Results:**

Of the 2,925 potential respondents, 906 completed the survey and were eligible for further analysis (response rate: 31%). Of these, 218 (24%) were prehospital EPs and 688 (76%) were EMTs and EMT-Ps, the latter two being significantly younger and less experienced. Almost half of the respondents reported having experience in caring for TIN patients as EMS responders (45% of EMTs/EMT-Ps vs 40% of prehospital EPs) but demonstrated significant gaps in non-medical and medical knowledge. Attitudes toward TIN patients were generally positive, but there were discrepancies between perceived comfort and actual communication behavior, with up to 25% of respondents overall avoiding questions they would ask non-TIN patients. Most respondents had no formal training in the appropriate management of TIN patients: only 7% of EMTs/EMT-Ps and 5% of prehospital EPs indicated having received such training during their professional or medical training. Our survey showed that 63% of EMTs/EMT-Ps and 62% of prehospital EPs agreed that there is an urgent need to increase awareness for TIN patients among EMS responders.

**Conclusion:**

Despite generally positive attitudes toward transexual, intersexual and non-binary patients, EMS responders in Germany demonstrated deficits in knowledge and clinical preparedness to care for this vulnerable patient population, indicating that the care of TIN patients has not yet become routine in EMS and highlighting a strong need for improved education and training.

## INTRODUCTION

The provision of prehospital emergency medical care requires healthcare professionals to address a wide range of medical needs in time-sensitive and often challenging situations and environments.^1^ Among the patient populations encountered in prehospital settings are individuals who identify as transgender, intersexual or non-binary (TIN).^2^ These individuals often face significant barriers when accessing healthcare due to discrimination, social stigma, and inadequate training of healthcare professionals.^3^,^4^ These barriers contribute to pronounced health disparities worldwide, with TIN patients at increased risk of mental health disorders, substance use disorders, and chronic health conditions.^5^,^6^ In both the United States (US) and Germany, TIN individuals represent approximately 0.6% of the adult population and up to 4.1% of adolescents, highlighting the need to address health disparities in this unique and growing population.^7^,^8^

Prehospital emergency medical services (EMS) or emergency departments (ED) are often the first point of contact between TIN patients and the healthcare system in emergencies.^9^ However, for many TIN individuals, this interaction is often accompanied by challenges, mainly due to the limited understanding of their specific healthcare needs by healthcare professionals.^10^ This deficit may lead to delays in care, misdiagnosis, and, in some cases, avoidance of emergency services by TIN patients altogether. Studies in the US and Germany/Europe show that nearly half of TIN individuals delay or avoid seeking emergency medical care because of concerns about mistreatment or previous negative experiences with healthcare professionals.^11^,^12^ There are multiple barriers to effective emergency medical care for TIN individuals, with the lack of education and training of emergency care professionals on gender diversity and the appropriate management of TIN patients being the key challenge.^13^ Surveys of emergency physicians (EP) and nurses working in EDs in Germany and the US show that although the majority have encountered TIN patients, most had significant knowledge gaps and reported feeling unprepared to provide competent care. Less than 30% of respondents stated they had received formal training in TIN care.^14^,^15^

Despite increasing awareness of these challenges, there remains a significant gap in the literature regarding the specific needs of TIN patients, particularly in the prehospital emergency care setting, as outlined in the 2024 Position Statement on Care for Transgender and Gender Diverse Prehospital Patients by the National Association of EMS Physicians (NAEMSP) in the US as well as in a recent scoping review on this topic by Farcas et al.^9^,^16^ Existing research has predominantly focused on hospital-based emergency care in the ED, with limited attention paid to EMS responders in prehospital settings.^2^ A recent survey of EPs and paramedics (EMT-Ps) in the US found that 94% of respondents expressed a willingness to include LGBTQ+ (lesbian, gay, bisexual, transgender, questioning/queer)-related content in their training programs, but few training institutions currently offer such training.^17^ Furthermore, there is a lack of data concerning the specific prehospital management of TIN patients in both the US and Europe. This gap is particularly concerning because the initial phase of emergency care is critical due to patients’ heightened vulnerability and anxiety, which are associated with uncertainty about the medical interventions to come.^9^

**Population Health Research Capsule**

What do we already know about this issue?

*Transgender, intersexual and non-binary (TIN) individuals experience significant health disparities and encounter barriers to care, including in emergency situations.*

What was the research question?

*We sought to explore the knowledge and attitudes of emergency medical services (EMS) responders in Germany regarding the care of TIN patients.*

What was the major finding of the study?

*EMS providers showed large gaps in knowledge about TIN care, but a strong desire to improve these findings.*

How does this improve population health?

*Recognizing the need for improved education and training of EMS responders in the management of TIN patients may promote more equitable emergency care.*

In this context, our goal in the present study was to explore the perspectives of EMS responders in Germany, including prehospital EPs, emergency medical technicians (EMT) and paramedics (EMT-P), regarding the care of TIN patients. While the involvement of prehospital EPs is specific to the Franco-German EMS system, EMTs and EMT-Ps are present in most EMS systems worldwide.^18^

## METHODS

### Study Design and Study Population

We conducted a cross-sectional study of EMS professionals in Germany including prehospital EPs (Notarzt), paramedics (Notfallsanitaeter), and EMTs (Rettungssanitaeter) using a web-based survey platform (SoSci Survey GmbH, Munich, Germany).^19^ Compared to the US EMS system, which relies primarily on EMTs and EMT-Ps to provide prehospital care, the German EMS system follows the Franco-German model, which involves a greater degree of physician involvement in prehospital care and emphasizes advanced medical interventions in the field rather than rapid transport to hospitals.^18^ To qualify as a prehospital EP, subspecialty training in prehospital emergency care and at least two years of clinical experience after medical school are required.^20^

The survey was distributed to 100 of approximately 1,000 randomly selected ambulance stations staffed with prehospital EPS across Germany, of which 65 agreed to distribute the survey to their EMS clinicians and responders. All active members of the prehospital EMS were considered eligible to participate. We estimated the number of prehospital EPs active as freelancers in the 65 ambulance stations to be approximately 975 and the number of EMTs and EMT-Ps to be 1,950, based on ambulance station staffing data. The Ethics Committee of Hannover Medical School approved the study (No. 10706_B0_K_2023).

### Survey Development

Survey development and administration is described in a prior publication.^14^ The survey instrument, which was developed by an interdisciplinary group of experts in questionnaire development and members of the TIN community in Germany, comprised 15 closed-ended items designed to assess participants’ experiences, attitudes, and education/training needs related to the care of TIN patients. These items were evenly distributed across three thematic categories: 1) five items related to experiences and knowledge; 2) five focused on respondents’ attitudes and 3) five on education/training needs. Response options were either single-choice or presented on a 4-point Likert scale (strongly agree – somewhat agree – somewhat disagree – strongly disagree). The survey instrument was already used in a previous study on EPs and nurses working in EDs in Germany.^14^ During the initial survey development, all survey items were tested for clarity with six EPs, five nurses, and eight EMS responders. To ensure the comparability of the two study samples for potential subsequent analysis, no adjustments were made to the ED staff survey. We collected the demographic parameters of the study participants and their informed consent for study participation and data use.

### Survey Administration

Participants had the opportunity to complete the anonymous, self-administered online survey during a four-week period in April 2023. Invitations were sent via e-mail to the medical directors of the ambulance stations with the request to distribute the survey link to their active staff members, including prehospital EPs, EMTs, and EMT-Ps. A reminder was sent after two weeks. All participants who met the inclusion criteria and completed at least the demographic parameters and the content category on experience were included in the final analysis.

### Primary Data Analysis

We extracted raw data from SoSci Survey into SPSS v 27 (SPSS Statistics, IBM Corp, Armonk, NY) and calculated standard descriptive statistics, including frequencies. The chi-square test was applied for group comparison. We considered *P* < 0.05 to be significant. The effect size Cramer’s V is interpreted as high (V=0.5), moderate (V=0.3) and low (V=0.1).

## RESULTS

During the study period, 958 surveys were returned, of which 906 were eligible for further analysis. Fifty-two participants did not complete at least the demographic parameters and the content category on experience and had to be excluded. Ninety-three percent of respondents completed all 15 items. The overall response rate was 31%, with approximately 975 prehospital EPs and 1,950 EMTs/EMT-Ps contacted by their respective ambulance station medical directors to participate in the study. Of the respondents, 218 (24%) were prehospital EPs and 688 (76%) were EMTs/EMT-Ps. There were significant intergroup differences in all demographic variables analysed (age, size of city of workplace, years of work experience) except for gender. In particular, EMTs/EMT-Ps were significantly younger (59% of EMTs/EMT-Ps ≤ 30y vs 8% of prehospital EPs ≤ 30y, *P*<0.001; V=0.441) and had fewer years of work experience than the EPs (69% of EMTs/EMT-Ps < 10 years vs 41% of prehospital EPs, *P*<0.001; V=0.277). Table 1 summarizes the characteristics of the respondents.

### Experience and Knowledge

**Figure 1:**
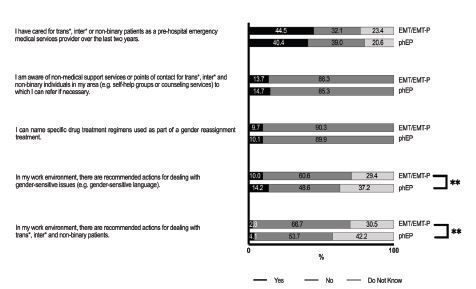
Experience and knowledge of pre-hospital emergency medical service providers in caring for transgender, intersexual and non-binary patients. ** = p <0.01 *phEP*, pre-hospital emergency physician; *EMT/EMT-P*, emergency medical technician or paramedic.

Almost half of respondents reported having cared for transgender and gender non-conforming patients as prehospital EMS professionals over the prior two years (Figure 1).

However, both prehospital EPs and EMTs/EMT-Ps demonstrated a lack of medical knowledge and awareness of non-medical support services for TIN individuals. Only 15% of prehospital EPs and 14% of EMTs/EMT-Ps reported being aware of non-medical support services or referral points for TIN people in their region, and only 10% of the respondents of both groups were able to name specific medication regimens associated with gender-affirming treatments. In addition, most prehospital EPs (96%) and EMTs/EMT-Ps (97%) reported that no official guidelines existed, and they were not aware of any recommendations for the management of TIN patients in the prehospital EMS setting.

### Attitudes

**Figure 2:**
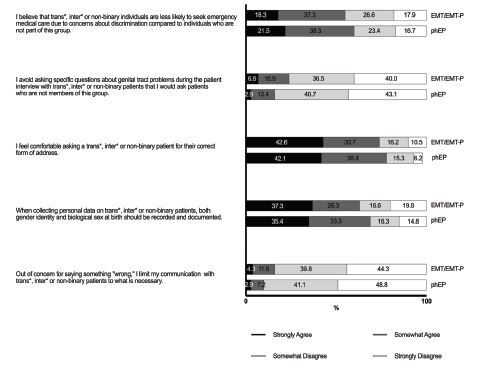
Attitudes of pre-hospital emergency medical service providers regarding transgender, intersexual, and non-binary patients. *phEP*, pre-hospital emergency physician; *EMT/EMT-P*, emergency medical technician or paramedic.

Most respondents in both groups agreed that both gender identity and biological sex at birth should be documented when collecting personal information in the prehospital EMS setting (69% of prehospital EPs and 64% of EMTs/EMT-Ps) (Figure 2).

Seventy-nine percent of prehospital EPs and 74% of EMTs/EMT-Ps felt comfortable asking TIN patients for their correct form of address. Most also reported that they would not avoid asking questions about genital tract problems that they would usually ask non-TIN patients, nor would they limit their communication with TIN patients to what was necessary out of concern for saying something wrong. In terms of perceptions of oppression, around 60% of both participant groups agreed that they believed TIN individuals were less likely to seek emergency medical care than non-TIN individuals because of concerns about discrimination (60% of prehospital EPs and 58% of EMTs/EMT-Ps).

### Education and Training Needs

**Figure 3:**
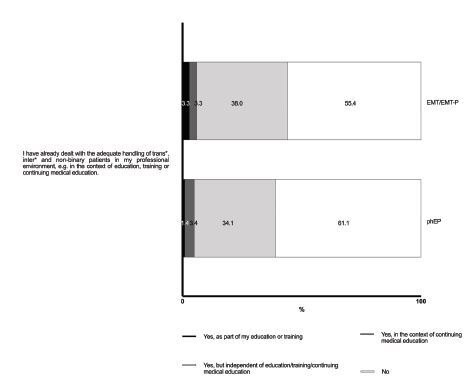
Education and training of pre-hospital emergency medical service providers regarding the care for transgender, intersexual and non-binary patients. *phEP*, pre-hospital emergency physician; *EMT/EMT-P*, emergency medical technician or paramedic.

Most respondents reported not having received any formal training in the appropriate management of TIN patients (61% of prehospital EPs and 55% of EMTs/EMT-Ps). Specifically, only 5% of the EPs and 7% of EMTs/EMT-Ps reported having received such training during their professional or medical education, including undergraduate, postgraduate, residency or fellowship programs. Thirty-four percent of prehospital EPs and 38% of EMTs/EMT-Ps reported learning about this topic on their own (Figure 3).

**Figure 4:**
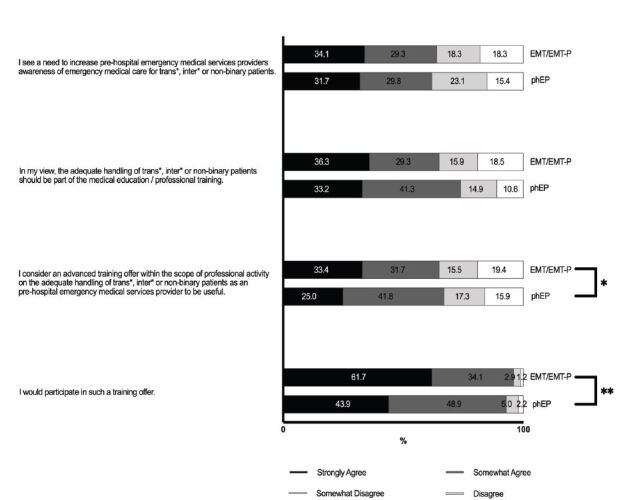
Education and training needs of pre-hospital emergency medical service providers regarding the care for transgender, intersexual and non-binary patients. * P < 0.05, ** P <0.01, *phEP*, pre-hospital emergency physician; *EMT/EMT-P*, emergency medical technician or paramedic.

A significant proportion of both groups, prehospital EPs (62%) and EMTs/EMT-Ps (63%), agreed with the statement that there is a need to raise awareness among EMS professionals regarding the care of TIN patients. In addition, 75% of prehospital EPs and 66% of EMTs/EMT-Ps felt that this should be included in medical education and professional training. Accordingly, more than 60% of participants in both groups expressed the belief that continuing medical education (CME) on the appropriate management of TIN patients would be valuable (67% of prehospital EPs and 65% of EMTs/EMT-Ps) and that they would participate in such CME if offered (93% of the EPs and 96% of EMTs/EMT-Ps) (Figure 4).

## DISCUSSION

In this cross-sectional survey, we found that although most EMS professionals in Germany felt comfortable communicating with TIN patients at first glance, there were still large gaps in knowledge about key aspects of emergency care for this underserved patient population and a strong desire for improved education and training to address these findings.

Although almost half of respondents in both groups reported encounters with TIN patients as prehospital EMS professionals in the prior 24 months, almost 90% demonstrated knowledge gaps in terms of lack of awareness of non-medical support services to which TIN patients can be referred, or specific medical regimens related to gender transition used by TIN patients. These findings are consistent with data from Chisolm, Straker et al and our group showing that both EPs and ENs working in EDs in the US and Germany had inaccurate knowledge about the care of TIN patients.^14^,^15^ With regard to EMTs and EMT-Ps, the lack of explicit educational content on LGBTQ care in EMS training programs, as recently shown by Jalali et al, and the lack of official guidelines for addressing gender-related issues, is likely to have contributed to these gaps and may result in inadequate or delayed care, exacerbating the inequalities faced by these individuals in emergency situations.^21^,^22^

Given its role as a pivotal entry point into the health care system for this vulnerable population, it is imperative to strengthen the capacity of EMS professionals to address the needs of these patients, who find themselves in a particularly precarious situation. In this regard, the NAEMSP has recently published a position statement on Care for Transgender and Gender Diverse Prehospital Patients in the US, which explicitly underscores the significance of gender-affirming care within the prehospital context. This statement clearly outlines the steps necessary to provide optimal care for these patients in emergency situations and has the potential to serve as a model for Europe.^9^

It is also plausible that affected individuals may perceive this lack of knowledge as insensitivity. Supporting this, several studies examining the experiences of transgender and gender non-conforming patients in ED settings have shown that more than half of these individuals avoid EDs primarily due to a perceived lack of clinician sensitivity and anticipated discrimination.^11^,^23^ There is no published data focusing on the experiences of TIN patients in the prehospital emergency care setting. Turning the tables and examining the attitudes of EPs and ENs toward the care of TIN patients in the ED, it was demonstrated that, contrary to published patient experiences, most ED staff felt comfortable communicating with TIN patients.^14^,^15^ Similar to these findings, the majority of respondents in this study of prehospital EMS professionals felt comfortable asking TIN patients for the correct form of address and agreed that both gender identity and biological sex should be documented. Nevertheless, up to 15% of respondents reported limiting communication with TIN patients out of concern for saying something wrong, and up to 25% of respondents avoided asking questions about genital tract problems that they would ask non-TIN patients. This discrepancy between perceived comfort and actual communication behaviours suggests a need for targeted training to improve competence and communication skills among EMS professionalvs.

Previous research suggests that TIN individuals often experience misgendering or questioning of their identity during emergency care encounters, leading to feelings of disrespect and mistrust toward healthcare professionals.^10^,^12^ Greater focus on communication skills, particularly the correct use of gender-sensitive language and respectful communication practices, therefore, seems necessary. Raising awareness of the oppression of TIN patients in emergency care seems equally important, as almost 40% of respondents in this study were unaware that TIN individuals are less likely to seek emergency medical care than non-TIN individuals due to concerns about discrimination. This awareness seems critical to removing barriers to healthcare access for TIN individuals.^24^

A key result of this study is the lack of formal training in TIN care among EMS professionals, which is likely to have contributed to the above findings. This is consistent with previous research, both in the US and Europe, indicating that training on sexual and gender minorities is often inadequate or absent in healthcare education.^25^,^26^,^27^ Notably, most participants agreed with the need to raise awareness of the care of TIN patients among EMS professionals and supported the inclusion of TIN-specific content in medical education and training. Such training should include both clinical knowledge, such as recognising the unique health risks of TIN individuals, and non-clinical aspects including respectful communication.

For EMT-Ps, Kruse et al developed a 70–90 minute mandatory, asynchronous, online training module on sexual and gender minority health in the prehospital setting, which led to a significant increase in allyship among cisgender, heterosexual-identified frontline paramedics in Canada.^28^ Although not specifically tailored to EMS professionals, the InTraHealth self-learning platform for healthcare professionals in Germany provides guidance on addressing and preventing discrimination against TIN people in healthcare settings.^29^ In addition, the development of standardised protocols, informed by both healthcare professionals and members of the TIN community, could improve the consistency and quality of care provided to TIN patients in emergency situations. Involving the TIN community in the development of these protocols could also ensure that the protocols are sensitive and meet the real needs of the TIN patient population.^30^

## LIMITATIONS

This study used an unvalidated survey instrument that relied on self-reported data from prehospital EPs and EMTs/EMT-Ps, which may have been subject to response bias, including social desirability bias. A positive selection bias cannot be excluded, as EMS medical directors, prehospital EPs or EMTs/EMT-Ps with a greater interest in this topic may have been more likely to participate in the study. Also, no information was available on non-respondents. Only eight participants identified as gender-diverse. Therefore, we cannot comment on their specific experiences and attitudes, which may differ from other EMS personnel and have not been explored in the literature. Finally, the relatively low estimated response rate of 31% and the limitation of the sample to prehospital EPs and EMTs/EMT-Ps in Germany may limit the generalisability of our findings to other countries or healthcare systems, although EMTs and EMT-Ps can be found in many EMS systems worldwide.

## CONCLUSION

This study provides evidence that there are significant gaps in the knowledge and clinical preparedness of EMS professionals to care for TIN patients in the prehospital emergency care setting. While attitudes toward TIN patients were generally positive, educational and structural barriers remain. Addressing these challenges through the integration of TIN healthcare topics, including gender-sensitive and respectful communication, into undergraduate and further training programs for healthcare professionals seems crucial to ensure that all patients, regardless of their gender identity, receive equitable, respectful, and competent care.

## Figures and Tables

**Table 1:** Demographics and descriptive characteristics of prehospital emergency medical service professionals.

Demographics	Prehospital EP		EMT/EMT-P	
	
	N	%	N	%
Total	218	24.1	688	75.9
Gender				
Male	139	63.8	456	66.3
Female	76	34.9	221	32.1
Diverse	1	0.5	7	1.0
No answer	2	0.9	4	0.6
Age group (years)				
≤ 20	0	0.0	41	6.0
21 – 30	17	7.8	362	52.6
31 – 40	104	47.7	161	23.4
41 – 50	52	23.9	79	11.5
> 50	45	20.6	45	6.5
Size of city of workplace (thousands)				
< 20,000	35	16.1	175	25.4
20,000 – 100,000	74	33.9	252	36.6
> 100,000 – < 1,000,000	82	37.6	203	29.5
≥ 1,000,000	27	12.4	58	8.4
Working experience (years)				
In training	0	0.0	51	7.4
< 5	27	12.4	196	28.5
5 – 10	63	28.9	230	33.4
> 10	128	58.7	211	30.7

*EP*, emergency physician; *EMT/EMT-P*, emergency medical technician or paramedic.
